# 3-(4-Bromo­phenyl­sulfon­yl)-5-cyclo­hexyl-2-methyl-1-benzofuran

**DOI:** 10.1107/S1600536812001791

**Published:** 2012-01-21

**Authors:** Hong Dae Choi, Pil Ja Seo, Uk Lee

**Affiliations:** a Department of Chemistry, Dongeui University, San 24 Kaya-dong Busanjin-gu, Busan 614-714, Republic of Korea; b Department of Chemistry, Pukyong National University, 599-1 Daeyeon 3-dong, Nam-gu, Busan 608-737, Republic of Korea

## Abstract

In the title compound, C_21_H_21_BrO_3_S, the cyclo­hexyl ring adopts a chair conformation. The 4-bromo­phenyl ring makes a dihedral angle of 80.88 (6)° with the mean plane of the benzofuran fragment. An intra­molecular C—H⋯O hydrogen bond is formed between an O atom of the sulfonyl group and one H atom of the aromatic ring such that a five-membered ring is formed. The crystal packing is stabilized by an inter­molecular C—H⋯O hydrogen bond, which links the mol­ecules into chains with graph-set notation *C*(6) running parallel to the *c* axis, and π–π stacking inter­actions [centroid–centroid distance = 3.6129 (12) Å].

## Related literature

For the biological activity of benzofuran compounds, see: Aslam *et al.* (2009 ▶); Galal *et al.* (2009 ▶); Khan *et al.* (2005 ▶). For natural products with benzofuran rings, see: Akgul & Anil (2003 ▶); Soekamto *et al.* (2003 ▶). For the crystal structures of related compounds, see: Choi *et al.* (2011 ▶); Seo *et al.* (2011 ▶). For puckering parameters, see: Cremer & Pople (1975 ▶). For hydrogen-bond motifs, see: Bernstein *et al.* (1995 ▶).
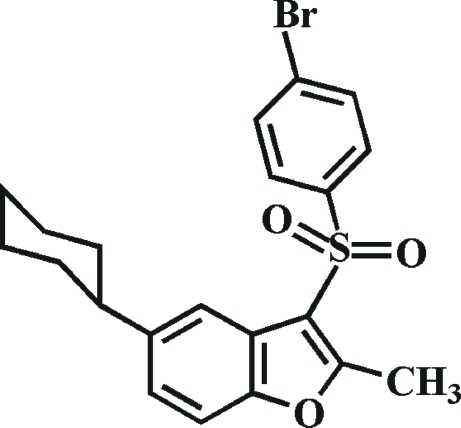


## Experimental

### 

#### Crystal data

C_21_H_21_BrO_3_S
*M* *_r_* = 433.35
Monoclinic, 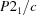

*a* = 16.8264 (3) Å
*b* = 8.7627 (1) Å
*c* = 13.3545 (2) Å
β = 104.248 (1)°
*V* = 1908.48 (5) Å^3^
*Z* = 4
Mo *K*α radiation
μ = 2.28 mm^−1^
*T* = 173 K
0.31 × 0.19 × 0.18 mm

#### Data collection

Bruker SMART APEXII CCD diffractometer
Absorption correction: multi-scan (*SADABS*; Bruker, 2009 ▶) *T*_min_ = 0.543, *T*_max_ = 0.686
18100 measured reflections
4739 independent reflections
3566 reflections with *I* > 2σ(*I*)
*R*_int_ = 0.031

#### Refinement

*R*[*F* ^2^ > 2σ(*F* ^2^)] = 0.038
*wR*(*F* ^2^) = 0.103
*S* = 1.03
4739 reflections
236 parameters
H-atom parameters constrained
Δρ_max_ = 0.60 e Å^−3^
Δρ_min_ = −0.71 e Å^−3^

### 

Data collection: *APEX2* (Bruker, 2009 ▶); cell refinement: *SAINT* (Bruker, 2009 ▶); data reduction: *SAINT*; program(s) used to solve structure: *SHELXS97* (Sheldrick, 2008 ▶); program(s) used to refine structure: *SHELXL97* (Sheldrick, 2008 ▶); molecular graphics: *ORTEP-3* (Farrugia, 1997 ▶) and *PLATON* (Spek,2009 ▶); software used to prepare material for publication: *SHELXL97*.

## Supplementary Material

Crystal structure: contains datablock(s) global, I. DOI: 10.1107/S1600536812001791/bx2395sup1.cif

Structure factors: contains datablock(s) I. DOI: 10.1107/S1600536812001791/bx2395Isup2.hkl

Supplementary material file. DOI: 10.1107/S1600536812001791/bx2395Isup3.cml

Additional supplementary materials:  crystallographic information; 3D view; checkCIF report

## Figures and Tables

**Table 1:** Hydrogen-bond geometry (Å, °)

*D*—H⋯*A*	*D*—H	H⋯*A*	*D*⋯*A*	*D*—H⋯*A*
C15—H15*A*⋯O2	0.98	2.40	3.125 (3)	131
C18—H18⋯O3^i^	0.95	2.48	3.120 (3)	125

Symmetry code: (i) 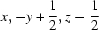.

**Fig. 1.**
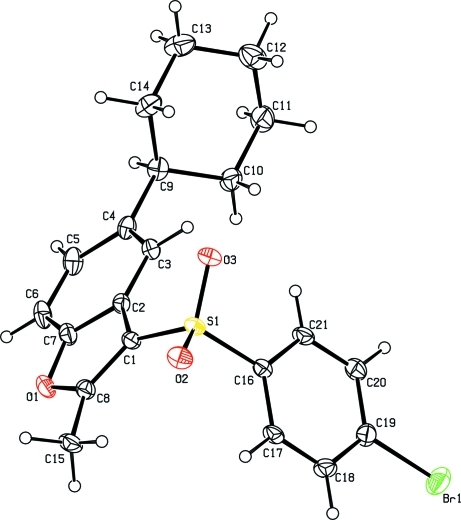
The molecular structure of the title compound with the atom numbering scheme. Displacement ellipsoids are drawn at the 50% probability level. H atoms are presented as small spheres of arbitrary radius.

**Fig. 2.**
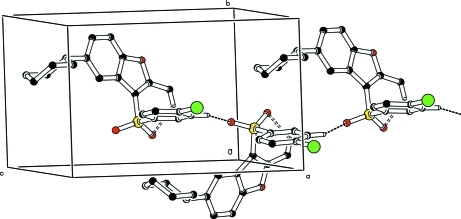
A view of the C—H···O interactions (dotted lines) in the crystal structure of the title compound. H atoms non-participating in hydrogen-bonding were omitted for clarity. [Symmetry codes: (i) x, - y + 1/2, z - 1/2; (iii) x, - y + 1/2, z + 1/2.]

**Fig. 3.**
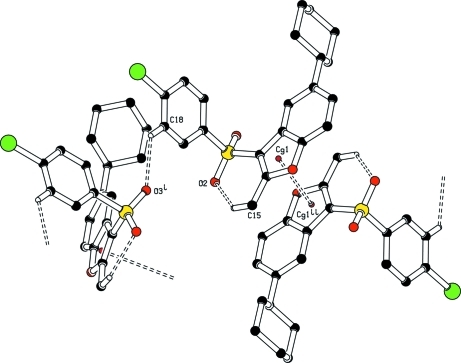
A view of the π–π interactions (dotted lines) in the crystal structure of the title compound. All H atoms were omitted for clarity. [Symmetry codes: (ii) - x, - y + 1, - z + 1.]

## supplementary crystallographic information

### Comment

Benzofuran derivatives have drawn much interest in view of their valuable biological properties such as antibacterial and antifungal, antitumor and antiviral, and antimicrobial activities (Aslam *et al.*, 2009, Galal *et al.*, 2009, Khan *et al.*, 2005). These benzofuran derivatives occur in a wide range of natural products (Akgul & Anil, 2003; Soekamto *et al.*, 2003). As a part of our ongoing study of 5-cyclohexyl-2-methyl-1-benzofuran derivatives containing either 3-(4-fluorophenylsulfonyl) (Choi *et al.*, 2011) or 3-phenylsulfonyl (Seo *et al.*, 2011) substituents, we report herein the crystal structure of the title compound. In the title molecule Fig. 1, the benzofuran unit is essentially planar, with a mean deviation of 0.006 (2) Å from the least-squares plane defined by the nine constituent atoms. The cyclohexyl ring is in the chair form as shown by the Cremer & Pople (1975) puckering parameters: Q = 0.560 (3)Å, θ = 1.8 (3)°, and φ = 187 (39) °]. The dihedral angle formed by the 4-bromophenyl ring and the mean plane of the benzofurn fragment is 80.88 (6)°. An intramolecular C–H···O hydrogen bond is formed between an O atom of the sulfonyl group and one H atom of the aromatic ring such that a five-membered ring is formed. The crystal packing is stabilized by an intermolecular C—H···O hydrogen bond, which links the molecules into chains with graph-set notation *C*(6) (Bernstein *et al.*, 1995) running parallel to c axis, Fig.2, Table 1 and π–π stacking interactions, Fig.3 , Table2.

### Experimental

77% 3-chloroperoxybenzoic acid (448 mg, 2 mmol) was added in small portions to a stirred solution of 3-(4-bromophenylsulfanyl)-5-cyclohexyl-2-methyl-1-benzofuran (361 mg, 0.9 mmol) in dichloromethane (30 mL) at 273 K. After being stirred at room temperature for 10h, the mixture was washed with saturated sodium bicarbonate solution and the organic layer was separated, dried over magnesium sulfate, filtered and concentrated at reduced pressure. The residue was purified by column chromatography (benzene) to afford the title compound as a colorless solid [yield 67%, m.p. 459–460 K; *R*_f_ = 0.51 (benzene)]. Single crystals suitable for X-ray diffraction were prepared by slow evaporation of a solution of the title compound in acetone at room temperature.

### Refinement

All H atoms were positioned geometrically and refined using a riding model, with C—H = 0.95 Å for aryl, 1.00 Å for methine, 0.99 Å for methylene and 0.98Å for methyl H atoms, respectively. *U*_iso_(H) =1.2*U*_eq_(C) for aryl, methine and methylene, and 1.5*U*_eq_(C) for methyl H atoms.

### Crystal data

**Table:**

C_21_H_21_BrO_3_S	*F*(000) = 888
*M**_r_* = 433.35	*D*_x_ = 1.508 Mg m^−^^3^
Monoclinic, *P*2_1_/*c*	Mo *K*α radiation, λ = 0.71073 Å
Hall symbol: -P 2ybc	Cell parameters from 5934 reflections
*a* = 16.8264 (3) Å	θ = 2.6–27.2°
*b* = 8.7627 (1) Å	µ = 2.28 mm^−^^1^
*c* = 13.3545 (2) Å	*T* = 173 K
β = 104.248 (1)°	Block, colourless
*V* = 1908.48 (5) Å^3^	0.31 × 0.19 × 0.18 mm
*Z* = 4	

### Data collection

**Table:**

Bruker SMART APEXII CCD diffractometer	4739 independent reflections
Radiation source: rotating anode	3566 reflections with *I* > 2σ(*I*)
graphite multilayer	*R*_int_ = 0.031
Detector resolution: 10.0 pixels mm^-1^	θ_max_ = 28.3°, θ_min_ = 1.3°
φ and ω scans	*h* = −18→22
Absorption correction: multi-scan (*SADABS*; Bruker, 2009)	*k* = −11→7
*T*_min_ = 0.543, *T*_max_ = 0.686	*l* = −17→17
18100 measured reflections	

### Refinement

**Table:**

Refinement on *F*^2^	Primary atom site location: structure-invariant direct methods
Least-squares matrix: full	Secondary atom site location: difference Fourier map
*R*[*F*^2^ > 2σ(*F*^2^)] = 0.038	Hydrogen site location: difference Fourier map
*wR*(*F*^2^) = 0.103	H-atom parameters constrained
*S* = 1.03	*w* = 1/[σ^2^(*F*_o_^2^) + (0.0533*P*)^2^ + 0.5992*P*] where *P* = (*F*_o_^2^ + 2*F*_c_^2^)/3
4739 reflections	(Δ/σ)_max_ = 0.001
236 parameters	Δρ_max_ = 0.60 e Å^−^^3^
0 restraints	Δρ_min_ = −0.71 e Å^−^^3^

### Special details

**Table:**

Geometry. All esds (except the esd in the dihedral angle between two l.s. planes) are estimated using the full covariance matrix. The cell esds are taken into account individually in the estimation of esds in distances, angles and torsion angles; correlations between esds in cell parameters are only used when they are defined by crystal symmetry. An approximate (isotropic) treatment of cell esds is used for estimating esds involving l.s. planes.
Refinement. Refinement of F^2^ against ALL reflections. The weighted R-factor wR and goodness of fit S are based on F^2^, conventional R-factors R are based on F, with F set to zero for negative F^2^. The threshold expression of F^2^ > 2sigma(F^2^) is used only for calculating R-factors(gt) etc. and is not relevant to the choice of reflections for refinement. R-factors based on F^2^ are statistically about twice as large as those based on F, and R- factors based on ALL data will be even larger.

### Fractional atomic coordinates and isotropic or equivalent isotropic displacement parameters (Å^2^)

**Table:**

	*x*	*y*	*z*	*U*_iso_*/*U*_eq_	
Br1	0.472627 (17)	0.39062 (4)	0.30549 (2)	0.05789 (12)	
S1	0.15069 (3)	0.28324 (5)	0.47083 (4)	0.02514 (12)	
O1	0.04011 (9)	0.67903 (17)	0.41683 (12)	0.0338 (4)	
O2	0.09589 (10)	0.18906 (17)	0.39712 (12)	0.0352 (4)	
O3	0.17627 (9)	0.23523 (16)	0.57671 (11)	0.0311 (3)	
C1	0.11029 (12)	0.4652 (2)	0.46944 (15)	0.0250 (4)	
C2	0.13778 (12)	0.5779 (2)	0.54926 (16)	0.0250 (4)	
C3	0.19440 (13)	0.5830 (2)	0.64531 (17)	0.0263 (4)	
H3	0.2256	0.4951	0.6720	0.032*	
C4	0.20449 (12)	0.7184 (2)	0.70136 (17)	0.0288 (5)	
C5	0.15718 (14)	0.8459 (2)	0.6599 (2)	0.0368 (5)	
H5	0.1644	0.9381	0.6985	0.044*	
C6	0.10060 (14)	0.8430 (2)	0.5654 (2)	0.0378 (5)	
H6	0.0691	0.9304	0.5385	0.045*	
C7	0.09228 (13)	0.7077 (2)	0.51260 (17)	0.0302 (5)	
C8	0.05259 (12)	0.5303 (2)	0.39189 (17)	0.0299 (5)	
C9	0.26505 (13)	0.7263 (2)	0.80598 (17)	0.0308 (5)	
H9	0.2646	0.8333	0.8320	0.037*	
C10	0.35212 (14)	0.6904 (3)	0.79958 (18)	0.0404 (6)	
H10A	0.3693	0.7654	0.7536	0.049*	
H10B	0.3534	0.5878	0.7690	0.049*	
C11	0.41288 (16)	0.6951 (3)	0.9066 (2)	0.0471 (6)	
H11A	0.4680	0.6648	0.8998	0.057*	
H11B	0.4166	0.8008	0.9335	0.057*	
C12	0.38692 (19)	0.5901 (3)	0.9823 (2)	0.0506 (7)	
H12A	0.4252	0.6013	1.0512	0.061*	
H12B	0.3895	0.4830	0.9597	0.061*	
C13	0.30039 (19)	0.6264 (4)	0.9898 (2)	0.0544 (7)	
H13A	0.2994	0.7293	1.0200	0.065*	
H13B	0.2835	0.5519	1.0362	0.065*	
C14	0.23995 (16)	0.6211 (3)	0.88381 (19)	0.0438 (6)	
H14A	0.1849	0.6511	0.8910	0.053*	
H14B	0.2363	0.5151	0.8574	0.053*	
C15	0.00326 (14)	0.4780 (3)	0.29081 (17)	0.0382 (5)	
H15A	0.0170	0.3717	0.2798	0.057*	
H15B	0.0151	0.5421	0.2361	0.057*	
H15C	−0.0551	0.4855	0.2894	0.057*	
C16	0.24026 (13)	0.3119 (2)	0.42616 (15)	0.0249 (4)	
C17	0.23543 (13)	0.3041 (2)	0.32088 (15)	0.0282 (4)	
H17	0.1846	0.2815	0.2737	0.034*	
C18	0.30421 (15)	0.3291 (3)	0.28488 (17)	0.0338 (5)	
H18	0.3012	0.3253	0.2130	0.041*	
C19	0.37760 (14)	0.3597 (3)	0.35501 (19)	0.0336 (5)	
C20	0.38350 (14)	0.3691 (3)	0.45991 (18)	0.0355 (5)	
H20	0.4346	0.3913	0.5067	0.043*	
C21	0.31415 (13)	0.3457 (3)	0.49569 (16)	0.0305 (5)	
H21	0.3170	0.3527	0.5675	0.037*	

### Atomic displacement parameters (Å^2^)

**Table:**

	*U*^11^	*U*^22^	*U*^33^	*U*^12^	*U*^13^	*U*^23^
Br1	0.03863 (18)	0.0885 (3)	0.0517 (2)	0.00045 (13)	0.02099 (14)	−0.00445 (14)
S1	0.0269 (3)	0.0250 (2)	0.0201 (2)	−0.0028 (2)	−0.0007 (2)	0.00023 (19)
O1	0.0247 (8)	0.0351 (8)	0.0382 (9)	0.0053 (6)	0.0014 (7)	0.0107 (7)
O2	0.0351 (9)	0.0351 (8)	0.0300 (8)	−0.0102 (7)	−0.0024 (7)	−0.0058 (7)
O3	0.0376 (8)	0.0300 (7)	0.0230 (8)	−0.0007 (6)	0.0019 (7)	0.0045 (6)
C1	0.0236 (10)	0.0275 (10)	0.0227 (10)	0.0008 (8)	0.0034 (8)	0.0042 (8)
C2	0.0218 (10)	0.0235 (9)	0.0308 (11)	0.0016 (7)	0.0087 (9)	0.0036 (8)
C3	0.0246 (11)	0.0238 (9)	0.0296 (11)	0.0018 (8)	0.0049 (9)	0.0003 (8)
C4	0.0234 (10)	0.0266 (10)	0.0379 (12)	−0.0027 (8)	0.0105 (9)	−0.0040 (9)
C5	0.0319 (12)	0.0247 (10)	0.0546 (15)	0.0010 (9)	0.0123 (12)	−0.0066 (10)
C6	0.0288 (12)	0.0266 (11)	0.0576 (16)	0.0083 (9)	0.0096 (12)	0.0052 (11)
C7	0.0211 (10)	0.0313 (11)	0.0377 (12)	0.0027 (8)	0.0065 (9)	0.0084 (9)
C8	0.0223 (10)	0.0365 (11)	0.0307 (11)	0.0000 (9)	0.0063 (9)	0.0084 (9)
C9	0.0303 (11)	0.0265 (10)	0.0347 (12)	−0.0040 (9)	0.0064 (10)	−0.0096 (9)
C10	0.0258 (12)	0.0608 (16)	0.0346 (13)	−0.0044 (11)	0.0075 (10)	−0.0037 (11)
C11	0.0308 (13)	0.0652 (18)	0.0415 (15)	−0.0023 (12)	0.0018 (11)	−0.0119 (13)
C12	0.0581 (18)	0.0480 (15)	0.0381 (15)	0.0051 (13)	−0.0026 (13)	−0.0055 (12)
C13	0.064 (2)	0.0674 (18)	0.0335 (14)	−0.0088 (15)	0.0159 (14)	−0.0057 (12)
C14	0.0414 (14)	0.0578 (16)	0.0359 (14)	−0.0108 (11)	0.0168 (12)	−0.0085 (11)
C15	0.0276 (11)	0.0559 (15)	0.0262 (11)	−0.0029 (11)	−0.0026 (9)	0.0109 (11)
C16	0.0267 (10)	0.0228 (9)	0.0228 (10)	0.0016 (8)	0.0013 (8)	−0.0004 (8)
C17	0.0287 (11)	0.0316 (11)	0.0191 (10)	0.0009 (9)	−0.0041 (9)	−0.0041 (8)
C18	0.0415 (13)	0.0364 (11)	0.0228 (11)	0.0034 (10)	0.0064 (10)	−0.0033 (9)
C19	0.0289 (12)	0.0365 (11)	0.0363 (13)	0.0022 (9)	0.0095 (10)	−0.0014 (10)
C20	0.0270 (12)	0.0450 (13)	0.0300 (12)	0.0003 (10)	−0.0018 (10)	−0.0028 (10)
C21	0.0314 (12)	0.0366 (11)	0.0195 (10)	0.0009 (9)	−0.0015 (9)	−0.0001 (9)

### Geometric parameters (Å, °)

**Table:**

Br1—C19	1.894 (2)	C10—H10B	0.9900
S1—O2	1.4330 (15)	C11—C12	1.509 (4)
S1—O3	1.4360 (15)	C11—H11A	0.9900
S1—C1	1.731 (2)	C11—H11B	0.9900
S1—C16	1.770 (2)	C12—C13	1.517 (4)
O1—C8	1.374 (3)	C12—H12A	0.9900
O1—C7	1.385 (3)	C12—H12B	0.9900
C1—C8	1.359 (3)	C13—C14	1.526 (4)
C1—C2	1.444 (3)	C13—H13A	0.9900
C2—C7	1.391 (3)	C13—H13B	0.9900
C2—C3	1.398 (3)	C14—H14A	0.9900
C3—C4	1.391 (3)	C14—H14B	0.9900
C3—H3	0.9500	C15—H15A	0.9800
C4—C5	1.405 (3)	C15—H15B	0.9800
C4—C9	1.515 (3)	C15—H15C	0.9800
C5—C6	1.381 (3)	C16—C21	1.388 (3)
C5—H5	0.9500	C16—C17	1.390 (3)
C6—C7	1.369 (3)	C17—C18	1.376 (3)
C6—H6	0.9500	C17—H17	0.9500
C8—C15	1.473 (3)	C18—C19	1.380 (3)
C9—C10	1.521 (3)	C18—H18	0.9500
C9—C14	1.525 (3)	C19—C20	1.382 (3)
C9—H9	1.0000	C20—C21	1.381 (3)
C10—C11	1.540 (3)	C20—H20	0.9500
C10—H10A	0.9900	C21—H21	0.9500
			
O2—S1—O3	119.59 (9)	C12—C11—H11B	109.3
O2—S1—C1	109.78 (10)	C10—C11—H11B	109.3
O3—S1—C1	107.58 (9)	H11A—C11—H11B	107.9
O2—S1—C16	107.91 (10)	C11—C12—C13	110.9 (2)
O3—S1—C16	107.43 (9)	C11—C12—H12A	109.5
C1—S1—C16	103.33 (9)	C13—C12—H12A	109.5
C8—O1—C7	107.22 (15)	C11—C12—H12B	109.5
C8—C1—C2	108.29 (18)	C13—C12—H12B	109.5
C8—C1—S1	126.89 (17)	H12A—C12—H12B	108.0
C2—C1—S1	124.67 (15)	C12—C13—C14	111.3 (2)
C7—C2—C3	119.29 (19)	C12—C13—H13A	109.4
C7—C2—C1	104.56 (18)	C14—C13—H13A	109.4
C3—C2—C1	136.16 (18)	C12—C13—H13B	109.4
C4—C3—C2	119.09 (19)	C14—C13—H13B	109.4
C4—C3—H3	120.5	H13A—C13—H13B	108.0
C2—C3—H3	120.5	C13—C14—C9	112.3 (2)
C3—C4—C5	119.0 (2)	C13—C14—H14A	109.1
C3—C4—C9	120.07 (18)	C9—C14—H14A	109.1
C5—C4—C9	120.93 (19)	C13—C14—H14B	109.1
C6—C5—C4	122.8 (2)	C9—C14—H14B	109.1
C6—C5—H5	118.6	H14A—C14—H14B	107.9
C4—C5—H5	118.6	C8—C15—H15A	109.5
C7—C6—C5	116.5 (2)	C8—C15—H15B	109.5
C7—C6—H6	121.7	H15A—C15—H15B	109.5
C5—C6—H6	121.7	C8—C15—H15C	109.5
C6—C7—O1	126.46 (19)	H15A—C15—H15C	109.5
C6—C7—C2	123.3 (2)	H15B—C15—H15C	109.5
O1—C7—C2	110.23 (18)	C21—C16—C17	120.5 (2)
C1—C8—O1	109.70 (18)	C21—C16—S1	120.10 (16)
C1—C8—C15	134.7 (2)	C17—C16—S1	119.43 (16)
O1—C8—C15	115.61 (18)	C18—C17—C16	120.06 (19)
C4—C9—C10	112.16 (18)	C18—C17—H17	120.0
C4—C9—C14	111.38 (18)	C16—C17—H17	120.0
C10—C9—C14	110.2 (2)	C17—C18—C19	118.9 (2)
C4—C9—H9	107.6	C17—C18—H18	120.6
C10—C9—H9	107.6	C19—C18—H18	120.6
C14—C9—H9	107.6	C18—C19—C20	121.9 (2)
C9—C10—C11	111.8 (2)	C18—C19—Br1	118.82 (18)
C9—C10—H10A	109.3	C20—C19—Br1	119.28 (18)
C11—C10—H10A	109.3	C21—C20—C19	119.1 (2)
C9—C10—H10B	109.3	C21—C20—H20	120.5
C11—C10—H10B	109.3	C19—C20—H20	120.5
H10A—C10—H10B	107.9	C20—C21—C16	119.6 (2)
C12—C11—C10	111.7 (2)	C20—C21—H21	120.2
C12—C11—H11A	109.3	C16—C21—H21	120.2
C10—C11—H11A	109.3		
			
O2—S1—C1—C8	−21.0 (2)	C7—O1—C8—C15	−178.98 (18)
O3—S1—C1—C8	−152.62 (18)	C3—C4—C9—C10	59.7 (3)
C16—S1—C1—C8	93.9 (2)	C5—C4—C9—C10	−121.0 (2)
O2—S1—C1—C2	164.01 (17)	C3—C4—C9—C14	−64.3 (3)
O3—S1—C1—C2	32.4 (2)	C5—C4—C9—C14	115.0 (2)
C16—S1—C1—C2	−81.09 (18)	C4—C9—C10—C11	−178.6 (2)
C8—C1—C2—C7	0.4 (2)	C14—C9—C10—C11	−53.9 (3)
S1—C1—C2—C7	176.20 (16)	C9—C10—C11—C12	55.4 (3)
C8—C1—C2—C3	−179.7 (2)	C10—C11—C12—C13	−55.3 (3)
S1—C1—C2—C3	−3.9 (4)	C11—C12—C13—C14	55.3 (3)
C7—C2—C3—C4	−0.7 (3)	C12—C13—C14—C9	−55.6 (3)
C1—C2—C3—C4	179.4 (2)	C4—C9—C14—C13	179.6 (2)
C2—C3—C4—C5	0.3 (3)	C10—C9—C14—C13	54.5 (3)
C2—C3—C4—C9	179.58 (18)	O2—S1—C16—C21	−155.16 (16)
C3—C4—C5—C6	0.1 (3)	O3—S1—C16—C21	−24.96 (19)
C9—C4—C5—C6	−179.2 (2)	C1—S1—C16—C21	88.60 (18)
C4—C5—C6—C7	0.0 (4)	O2—S1—C16—C17	26.52 (19)
C5—C6—C7—O1	−179.5 (2)	O3—S1—C16—C17	156.72 (16)
C5—C6—C7—C2	−0.5 (3)	C1—S1—C16—C17	−89.71 (17)
C8—O1—C7—C6	178.8 (2)	C21—C16—C17—C18	0.4 (3)
C8—O1—C7—C2	−0.3 (2)	S1—C16—C17—C18	178.71 (16)
C3—C2—C7—C6	0.9 (3)	C16—C17—C18—C19	0.8 (3)
C1—C2—C7—C6	−179.2 (2)	C17—C18—C19—C20	−1.4 (3)
C3—C2—C7—O1	−179.99 (18)	C17—C18—C19—Br1	178.92 (16)
C1—C2—C7—O1	−0.1 (2)	C18—C19—C20—C21	0.6 (3)
C2—C1—C8—O1	−0.6 (2)	Br1—C19—C20—C21	−179.67 (17)
S1—C1—C8—O1	−176.28 (15)	C19—C20—C21—C16	0.6 (3)
C2—C1—C8—C15	178.8 (2)	C17—C16—C21—C20	−1.2 (3)
S1—C1—C8—C15	3.1 (4)	S1—C16—C21—C20	−179.45 (17)
C7—O1—C8—C1	0.5 (2)		

### Hydrogen-bond geometry (Å, °)

**Table:**

*D*—H···*A*	*D*—H	H···*A*	*D*···*A*	*D*—H···*A*
C15—H15A···O2	0.98	2.40	3.125 (3)	131
C18—H18···O3^i^	0.95	2.48	3.120 (3)	125

Symmetry codes: (i) *x*, −*y*+1/2, *z*−1/2.

### Table 2 π-π stacking interaction in (I)

Cg1 is the centroid of the O1/C7/C2/C1/C8 ring, φ is the dihedral angle (°) between the planes of the rings, d is the distance (Å) between the ring centroids and Δ is the displacement (Å) of the centroid of ring 2 relative to the intersection point of the normal to the centroid of ring 1 and the least-squares plane of ring 2

**Table:**

Ring 1	Ring 2	φ	d	Δ
O1/C7/C2/C1/C8	(O1/C7/C2/C1/C8)^ii^	0.0	3.6129 (12)	0.938

Symmetry code: (ii)-x,1-y,1-z
